# Decision-making in district health planning in Uganda: does use of district-specific evidence matter?

**DOI:** 10.1186/s12961-019-0458-6

**Published:** 2019-06-06

**Authors:** Dorcus Kiwanuka Henriksson, Stefan Swartling Peterson, Peter Waiswa, Mio Fredriksson

**Affiliations:** 1 Karolinska Institutet, Stockholm and Uppsala University, Uppsala, Sweden; 2 United Nations Children’s Fund, New York, Karolinska Institutet, Stockholm, Uppsala University, Uppsala, Sweden; 3 School of Public Health, Kampala and Karolinska Institutet, Makerere University College of Health Sciences, Stockholm, Sweden; 4 Department of Public Health and Caring Sciences, Uppsala University, Uppsala, Sweden

**Keywords:** District health system, planning, decision-making, evidence, work plans, Uganda

## Abstract

**Background:**

In a decentralised health system, district health managers are tasked with planning for health service delivery, which should be evidence based. However, planning in low-income countries such as Uganda has been described as ad hoc. A systematic approach to the planning process using district-specific evidence was introduced to district health managers in Uganda. However, little is known about how the use of district-specific evidence informs the planning process. In this study, we investigate how the use of this evidence affects decision-making in the planning process and how stakeholders in the planning process perceived the use of evidence.

**Methods:**

A convergent parallel mixed-methods study design was used, where quantitative data was collected from district health annual work plans for the financial years 2012/2013, 2013/2014, 2014/2015 and 2015/2016 as well as from bottleneck analysis reports for 2012, 2013, 2014 and 2015. Qualitative data was collected through semi-structured interviews with key informants from the two study districts.

**Results:**

District managers reported that they were able to produce more robust district annual work plans when they used the systematic approach of using district-specific evidence. Approximately half of the prioritised activities in the annual work plans were evidence based. Procurement and logistics, training, and support supervision activities were the most prioritised activities. Between 4% and 5.5% of the total planned expenditure was for child survival, of which 47% to 94% was from donor and other partner contributions.

**Conclusion:**

District-specific evidence and a structured process for its use to prioritise activities and make decisions in the planning process at the district level helped systematise the planning process. However, the reported limited decision and fiscal space, inadequate funding and high dependency on donor funding did not always allow for the use of district-specific evidence in the planning process.

## Background

The health sector in Uganda has undergone several changes and reforms since the Harare Declaration, which introduced the district health system (DHS) [1] and the introduction of decentralisation in its current form in 1997 [2]. Planning, which is one of the core activities within the DHS, has been affected, during this period, by these policy changes and met practical hindrances such as political and technical resistance to effective decentralisation, a rapid increase in the number of districts [3], limited financial resources and decision space [4], and implementation of vertical programmes [5, 6], to mention just a few. The weakened capacity of the Ministry of Health (MoH) due to inadequate number of staff to effectively coordinate, support and supervise the growing number of districts and absence of a regional level, have also affected the planning process [7].

In 1997, Uganda took on political, administrative and fiscal decentralisation, thereby transferring administrative, political and fiscal authority from the central government to the local government authorities, mainly in the form of devolution [2, 8]. Unlike many other countries, Uganda has no functional ‘intermediate level’ such as provinces or regions [9], although this level has been planned for and included in the health sector development plan 2015/2016–2019/2020 [10]. The District Council composed of elected officials is the highest political authority in the district and makes the final decision on approval of the annual district health work plan. The administrative and technical team at the district is divided into directorates, including for health, which are responsible for developing annual work plans [2].

**Fig. 1:**
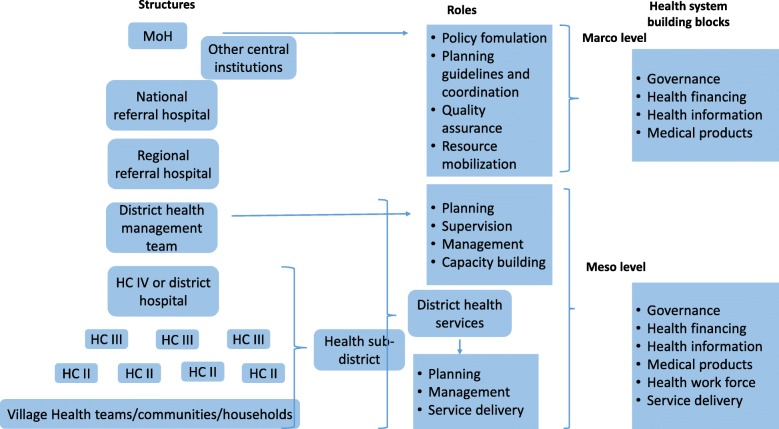
Managerial organisation of the health sector in Uganda

The provision of healthcare is the responsibility of two levels of government – the central (macro) and the local (meso and micro) levels (Fig. 1). The DHS is part of the district local government [2] and is a self-contained segment of the national health system. The DHS is responsible for the delivery of health services, planning, management and implementation of policies [7].

Although planning should be evidence based to prioritise activities [11, 12], in low-income countries (LICs) such as Uganda, priority-setting has been described as ad hoc [13]. It has been documented that health managers make decisions based on national and donor priorities and, in some cases, based on previously funded activities [14–17]. This situation has been attributed to, amongst other factors, the lack of tools to aid priority-setting and decision-making [16, 17]. Even when tools are available, they are not always used by decision-makers in LICs as they lack credibility for priority-setting in low-resource settings [18, 19]. Many of these tools have been tested in pilot settings and do not evaluate the impact on actual priority-setting [19].

Similarly, in Uganda, the lack of tools to assess performance in the DHS prevents the identification of gaps in service delivery and the definition of priorities for actions to bridge the gaps during the annual planning process [16]. A systematic approach of using district-specific evidence in the planning process was introduced to the district health management teams (DHMT) in 21 districts, by the Community and District Empowerment for Scale-up (CODES) project. The CODES project targeted key interventions to reduce child deaths due to diarrhoea, pneumonia and malaria, for which the planning processes focused on child survival. Results on the effect of the interventions on child survival are being finalised. A specific focus of the project was to build the capacity of the DHMT to use district-specific evidence in the planning process [20].

District-specific evidence in this study refers to the data that was collected through routine Health Management Information Systems and other surveys, for example, Lots Quality Assurance Surveys. Evidence also includes information from community dialogues with care givers of children under 5 years of age, health workers and village health teams. The structured process refers to how this evidence was analysed by the DHMT to identify gaps in service delivery and prioritise solutions to overcome those gaps [20].

The use of evidence in the field of medicine is usually understood as evidence-based medicine, which is defined as “*the conscientious, explicit, and judicious use of current best evidence in making decisions about the care of individual patients*” [21, 22]. Over the years, evidence-based health policy-making has become increasingly common [23, 24], thus shifting the focus from the individual level to the population level [25].

What constitutes ‘evidence’ in the area of evidence-based and evidence-informed policy-making has been subject of discussion. Rychetnik et al. [26] define evidence as facts or testimony in support of a conclusion, statement or belief. Oxman et al. [27] have a similar definition, saying that evidence is concerned with actual or asserted facts intended for use to support a conclusion. However, both of these definitions are broad and do not speak of the context within which evidence is used, what is considered evidence and who uses the evidence [26] .

Decisions are not made solely based on evidence, but other factors are considered as well, such as the priorities at the time the policy is decided, the context and financial resources, and the actors involved [28]. Therefore, the use of evidence in policy-making involves a complex process of interactions between policy actors and different powers, interactions and agendas [28], and can be affected by institutional characteristics and the political process.

Evidence-based planning, as defined by Steen [29], is a process of basing decisions about ways to address a problem on information to achieve the best results. Although evidence-based planning is not as commonly referred to in the literature, it follows similar principles as evidence-based or evidence-informed policy-making, with the primary purpose being the use of evidence to inform decision-making.

The CODES project introduced the Tanahashi bottleneck analysis tool that enabled the DHMT to identify bottlenecks to implementation of key interventions for diarrhoea, pneumonia and malaria. Casual analysis was done guided by a management checklist. Solutions were later subjected to a prioritisation matrix that had a scale of 1 to 3 in the areas of supporting evidence, policy, capacity, affordability, acceptability and equity. Examples of bottlenecks were loss to follow-up and high dropout rate for routine immunisation and inadequate utilisation of malaria case management services. Details on the CODES project is described elsewhere [16, 30–32].

This process of identification of bottlenecks and prioritisation of solutions was conducted each year for each of the districts [16, 20] and documented in a bottleneck analysis report. The districts then planned for activities during the district annual planning process, based on the analysis of district-specific evidence, resulting in a district health annual work plan. The district health annual work plan is a document that gives a detailed account of how the district proposes to accomplish health service delivery [33].

The tools introduced by the CODES project to facilitate the use of district-specific evidence in the planning process were appreciated and adopted into the planning process. However, there were barriers such as a perceived lack of decision and fiscal space, politicians with their own priorities, gaps in human resources and inadequate health information systems [4, 34]. Another challenge was the central or national level priority-setting [7, 33, 35].

The MoH in Uganda, with the experience gained from the CODES project, is advocating for the use of district-specific evidence in the district planning process [33]. Thus, the MoH incorporated use of district-specific information in the new planning guidelines that are being rolled out at the district level [33]. However, little is known about how the use of district-specific evidence informs the planning process. For instance, what kind of priorities end up in the district health annual work plans after gaps have been identified? How does central priority-setting affect the district planning process? Therefore, the aim of the study was to investigate to what extent district-specific evidence informed prioritisation of child survival activities in the annual district work plans and how stakeholders in the planning process perceived the use of evidence. Results from this study will contribute to the body of knowledge on the use of district-specific evidence in the district planning process.

## Methods

### Study site

The study was conducted in two purposively selected districts in Uganda [36], chosen because they were introduced to a systematic approach which utilised tools to facilitate the use of district-specific evidence in the planning process [20] and had used the approach for over 4 years. One of the districts in the study was established in 2010 and has a population of approximately 150,000 people. For this study, it is referred to as district A. District B has a population of approximately 300,000 people and was established in the 1990s. Both districts are mainly rural, with approximately 60% of the people in both districts aged 18 years and under. See details in Table 1.

**Table 1:** District demographics

Demographics	District A	District B
Approximate^a^ total population	300,000	150,000
Year of creation	1990	2010
Approximate rural population	200,000	140,000
Approximate urban population	100,000	10,000
Number of health facilities	20	14

^a^Approximates were used to keep districts anonymous

### Study design and data collection

**Fig. 2:**
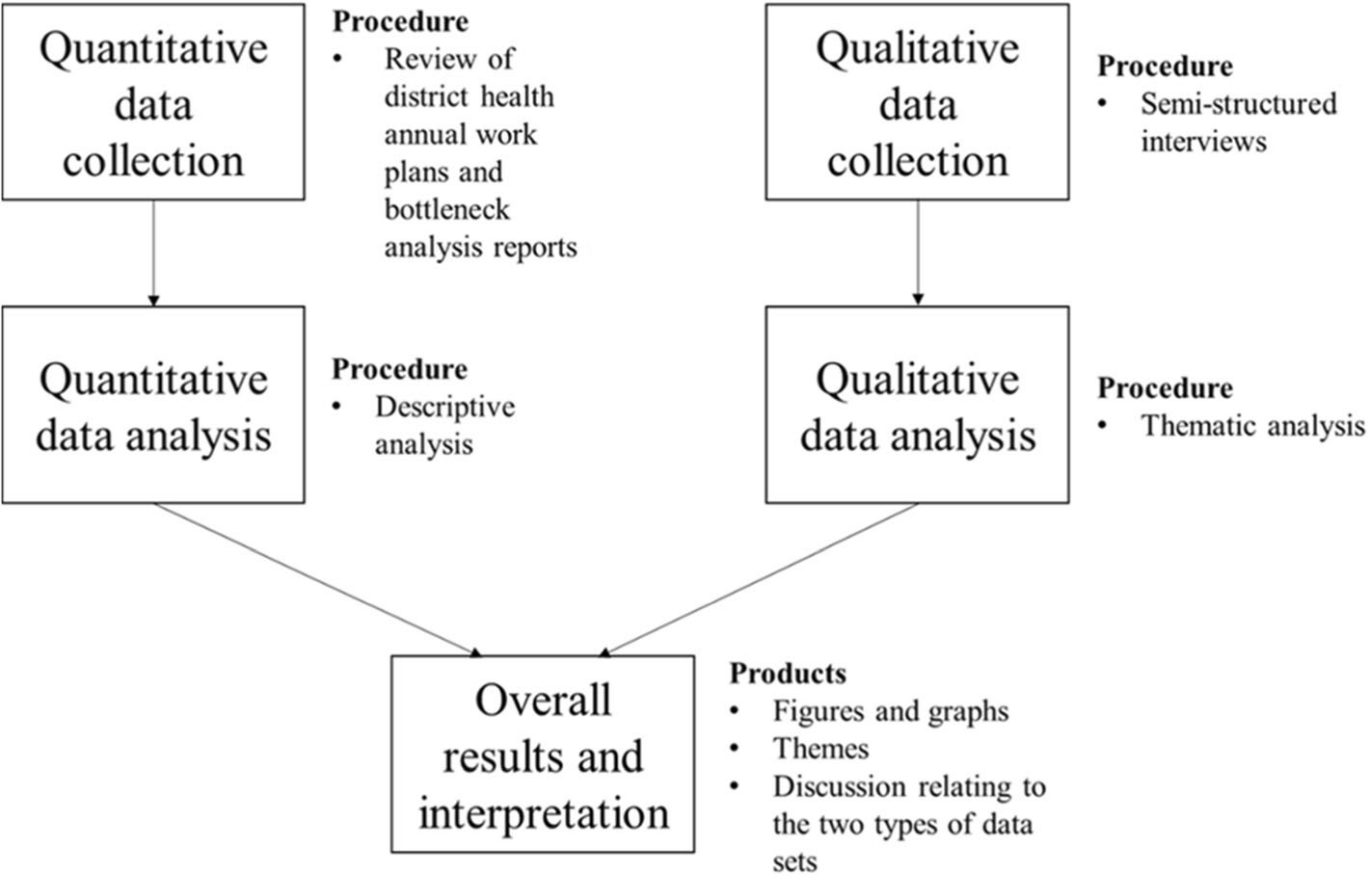
Study procedure

A convergent, parallel, mixed-methods design was used. Qualitative and quantitative data were collected separately within the same period and analysed separately, and the results were converged during interpretation [37, 38] (see Fig. 2 for the study procedure). This study design was used because it allowed for different but complementary data collection on the same topic and merging of results during interpretation, which facilitated a broader understanding of how district-specific evidence informed decision-making in the planning process [39, 40].

Quantitative information was collected from the district health annual work plans for the financial years 2012/2013, 2013/2014, 2014/2015 and 2015/2016 as well as from bottleneck analysis reports for 2012, 2013, 2014 and 2015, which were outputs of the bottleneck analysis process. To further understand how the use of local evidence affected the planning process and its perceived benefits, qualitative information was collected using semi-structured interviews with eight key informants [41, 42], four from each district. The key informants were purposefully selected [36] due to their involvement and knowledge of the district planning process. The interview guide used for the semi-structured interviews was developed based on the WHO decentralisation analysis framework [43] (Table 2). The framework was used because it considers the background of decentralisation and organisational processes and systems in the health sector under decentralisation. At the same time, the framework takes into account the difficulty of establishing direct casual links to changes within the health system. The framework also emphasises the need to search for alternative explanations for changes that take place in the health system [43].

**Table 2:** Interview guide for key informant interviews

Objective/theme	Question
Understanding of evidence and its purpose	1. From your perspective, what do you understand by evidence?
	2. What benefits would you hope to receive by using evidence?
	3. In what ways has the use of evidence met your expectations?
	4. In what ways has using evidence failed to meet your expectations?
Perceived outcomes of using local evidence in the planning process	1. Have you been using local evidence to meet your specific needs?
	2. Have you been using local evidence to meet your specific needs in the planning process?
	3. Do you think that the use of local evidence has benefited you and the district? If yes:
	4. In what way has the use of local evidence benefited you, the planning process and the district?
	5. Do you think that the use of local evidence will (or has already) had an impact on the district health system and the planning process?
	6. In your opinion, what has been the most important outcome of using local evidence in the planning process?
How the use of locally generated data has affected the planning process and resource allocation for child survival activities	1. To what extent has the use of local evidence affected your role in the planning process?
	2. Were any activities that were not previously identified as priorities included in the annual work plan?
	3. Has the use of local evidence affected your authority in the planning process?
	4. Have you had any professional or political concerns regarding the use of local evidence OR have there been? If yes, elaborate.
	5. Has the use of evidence affected the nature and range of resources allocation decisions made in the district?
	6. How much freedom do you or other local managers have to reallocate funds between line items and programmes?

### Study team

The research team consisted of two Ugandan Public health specialists (DKH and PW) with experience as heads of a DHT, a Swedish health systems specialist (SSP) with previous experience working in Uganda, and a Swedish researcher in health and political reforms (MF). No one on the team was working within the district health system.

### Analysis

Quantitative and qualitative data analyses were performed independently of each other. For the quantitative data, a descriptive analysis of the district health annual work plans and bottleneck analysis reports was conducted [44]. The bottleneck analysis was to establish which child survival activities were included in the work plans concerning the identified bottlenecks and how these activities were financed. Results were presented in graphs and figures. Thematic analysis [45–47] was conducted on the quantitative data to classify responses within themes related to use of district-specific evidence in the planning process and allocation of financial resources.

Budgets for wages and medicines were excluded from the budget analysis. It was not possible to estimate, by analysing the annual health work plans, how much time each employee devoted to child survival activities each year or the district expenditure on medicines related to child survival.

## Results

### Inclusion of proposed actions to identified gaps in the district health annual work plans

About half of the activities defined during the bottleneck and casual analysis process were included in the annual health work plan. The number of proposed activities increased by 13 and 10 from 2012/2013 to 2015/2016 in districts A and B, respectively. However, the proportion of the defined solutions that were included in the district annual health work plan was about the same over the 4-year period. In district A, 57% of identified solutions were included in the annual work plan in 2012/2013 and 60% in 2015/2016. In district B, 100% of identified solutions were prioritised in the annual health work plan in 2012/2013 and only 40% in 2015/2016.

The district managers also reported the inclusion of identified priorities into the work plans. However, they acknowledged that activities, even if included in the work plans, could not always be implemented because they sometimes did not have the power to take the necessary decisions themselves. One of the managers said:

“*We are understaffed, but we do not have that power to say, ‘if the staff is not enough let us recruit more.’ So, the evidence is there that we lack staff, but there is no way we can fill that gap because we are not in that position to do so.*” Manager, district A

Activities that either had no direct financial implications or were considered to have very high financial implications were not always included in the annual health work plans, for example, procurement of expensive equipment.

“*Evidence can help us plan, but again, plans must be backed up with a budget or money. So we only plan within the expected budget. Those* [activities] *that are expensive we cannot plan for them, we keep on postponing them.*” Manager, disrict B

### Child survival activities prioritised in the district health annual work plans

A wide range of child survival activities were prioritised in the district health annual work plans. For purposes of this analysis, activities were categorised into (1) support supervision, (2) planning/meetings, (3) mobilisation and advocacy, (4) data-related activities, (5) training, (6) logistics and procurement, and (7) immunisation outreach. These categories were used because they represented all child survival activities in the district health annual work plans (see Table 3 for a description of the categories).

**Table 3:** Categories used for the work plan analysis

Categories	Description
Support supervision	All support supervision activities related to child survival interventions irrespective of who the supervisor or supervisee was
Planning/meetings	All meetings related to work planning activities
Mobilisation and advocacy	Activities related to providing information and advocating for child survival interventions
Data	Activities related to data collection for child survival activities
Training/mentorship	Training activities related to the provision of child survival services
Logistics and procurement	Activities related to reproduction, purchase and distribution of child survival-related commodities apart from procurement of medicines
Outreaches for immunisation	Immunisation outreaches conducted

**Fig. 3:**
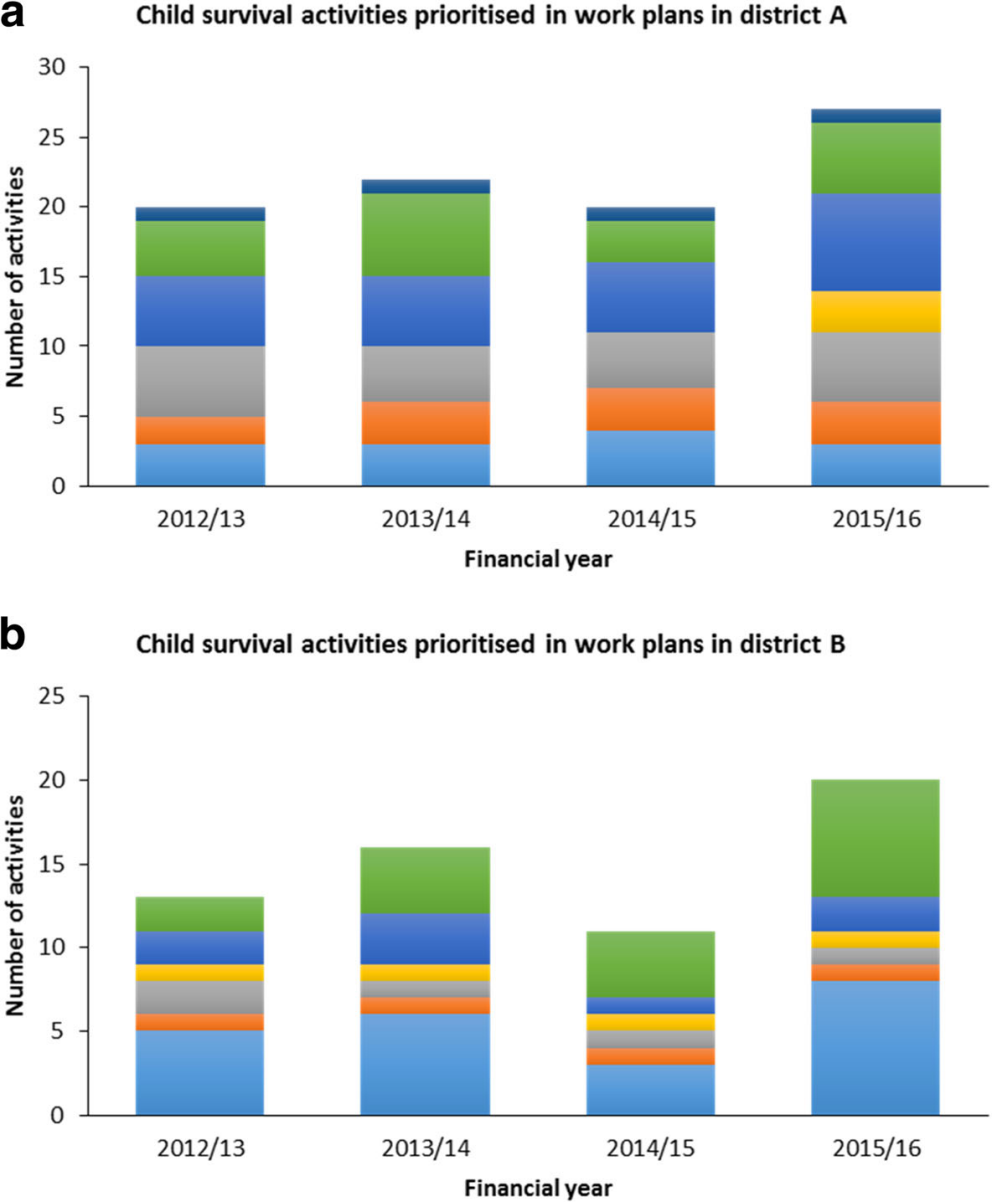
Child survival activities included in the district annual health work plans for districts **a** and **b**

Figure 3 shows that the majority of activities in the work plans were in the categories of logistics and procurement, support supervision, and training.

**Fig. 4:**
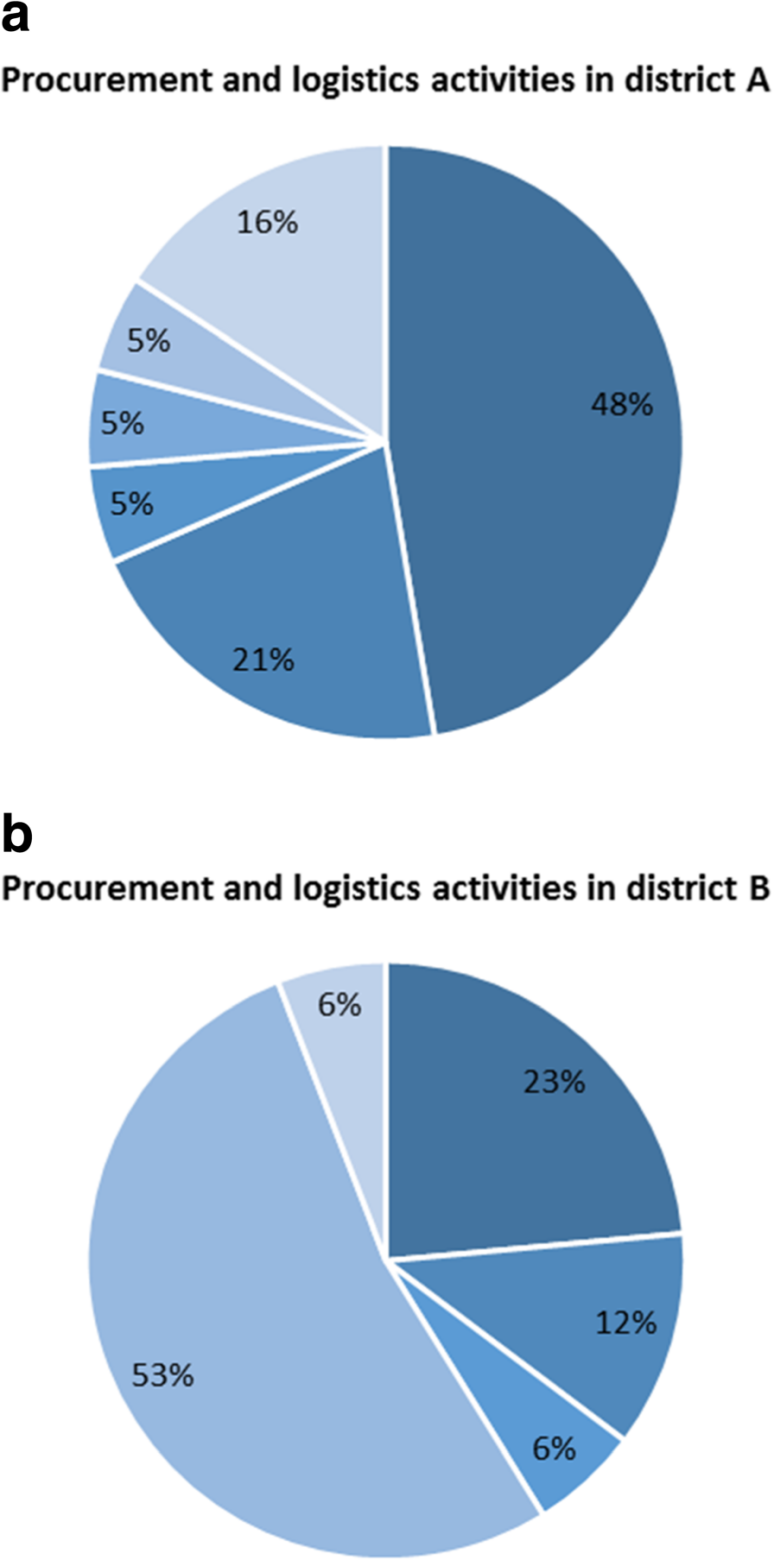
Planned activities for procurement and logistics for 2012–2016 for districts **a** and **b**

Further analysis of the procurement and logistics category, which accounted for the majority of the planned activities, showed variation in the kind of activities that were prioritised in each of the districts (Fig. 4). This variation in activities could have resulted in decisions being informed by district-specific evidence.

### Funding for child survival activities

**Fig. 5:**
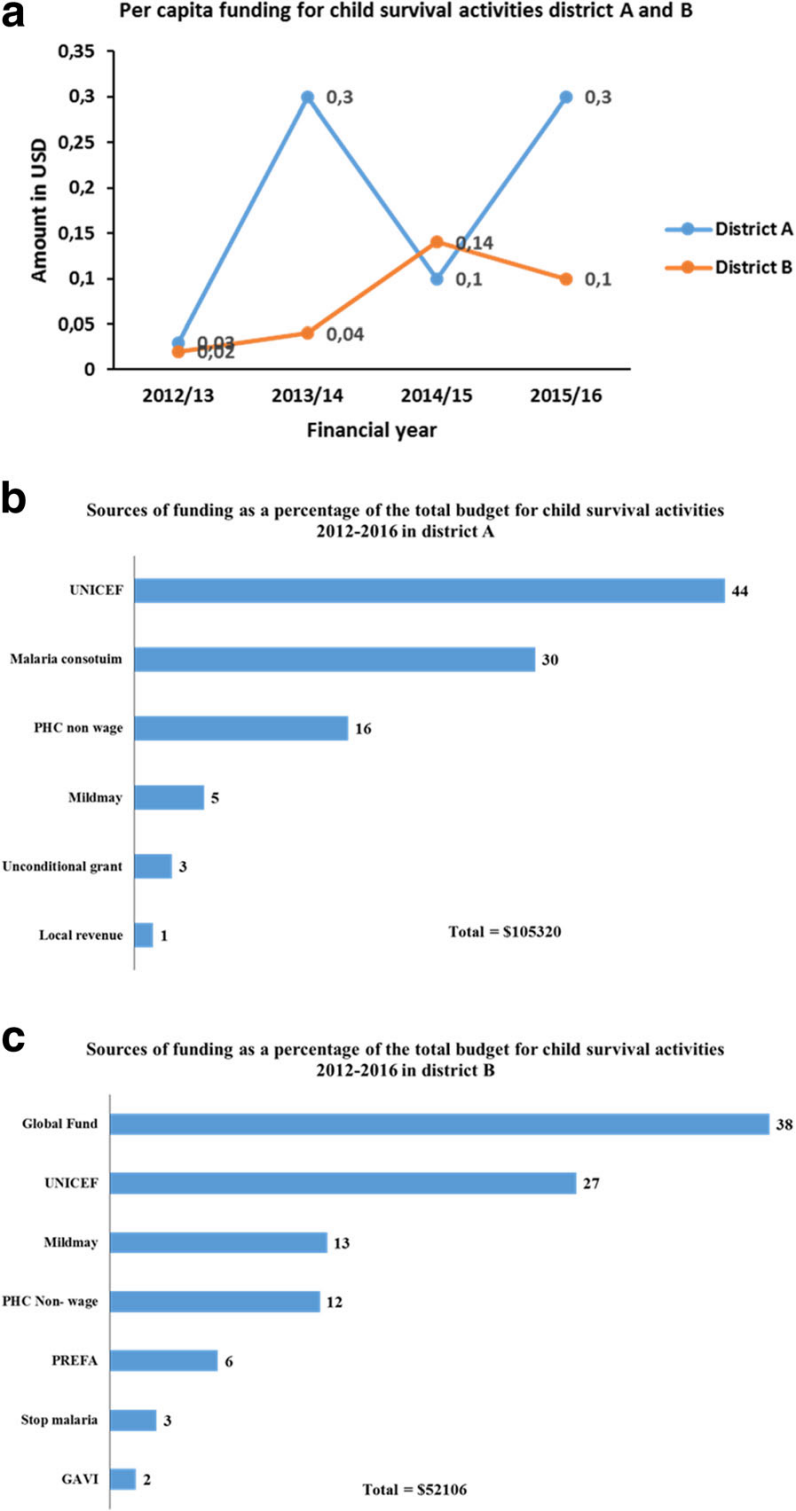
Planned per capita funding for child survival activities **a**, and sources of funding (%) for 2012-2016 **b** and **c**

There was an increase in the planned budget for child survival activities between 2012/2013 and 2015/2016 in both districts. In district A, the planned expenditure increased from US$ 4550 to US$ 45,185, while in district B it rose from US$ 6626 to US$ 28,327. Child survival activities accounted for between 4% and 5.5% of the total planned expenditure on health services with per capita funding of US$ 0.3 and US$ 0.1 in districts A and B, respectively (Fig. 5a).

Although funding for child survival activities increased over the 4-year period, the levels were still low, and one of the district managers emphasised this:

“*The funding is very, very little when you look at what we have planned and what has come as a contribution from the PHC* [primary healthcare]*, and funds are minimal.*” Manager, district A

### Sources of funding for child survival activities

There was a variation in the sources of funding over the four financial years (Fig. 5b, c). Government funding for child survival activities over the 4-year period, on average, accounted for 16% and 12% in district A and B, respectively. During the same period, donors and other partners contributed most of the funding for child survival activities, ranging from 47% to 94%. In district A, over the 4-year period, UNICEF provided the most significant proportion of funding for child survival activities (44%), while in district B, the Global Fund for HIV/AIDS, Tuberculosis and Malaria provided 38% of the funding. Local government revenue (local revenue) accounted for the least amount of funding for child survival activities.

Results from both districts showed that the districts were dependent on partner/donor funding for child survival activities, although donors sometimes had their own priorities that were not necessarily those informed by the district-specific evidence. For example, the geographical area of implementation of programmes may not necessarily be in the areas that have the most need. The managers also expressed dependence on partner funding:

“*So, we still feel that the money is little and if we had no partners, I think we would not be doing anything.*” Manager, district B

District managers reported that they were further constrained by the difficulty in reallocating resources as the funding came with guidelines for its use and could not be easily reallocated.

“*If they* [funds] *are conditional, they have guidelines that come with those funding; they are not supposed to be reallocated, they are supposed to be used for whatever activities that they are supposed to do. If it is immunisation, it must be immunisation, not any other thing.*” Manager, district A

### Outcomes of using local evidence in the planning process

District managers reported that the use of district-specific evidence in planning was minimal before the introduction of evidence-based planning by the CODES project. They also stated that they were able to prioritise activities backed by evidence and produce more robust work plans. For instance, one of them said:

“*I must say the work plans we have now are more real and genuine and factual. Using data has enabled us to have realistic work plans.*” Manager, district A

The managers also reported having fewer disagreements with elected officials when evidence was used in the planning process, which made their work easier than before. DHMT members also said that using district-specific evidence encouraged dialogue and better teamwork and allowed them to think outside the box for innovative solutions to identified problems.

“*I think the most important* [outcome] *is that the process has inculcated in us the habit of discussion, of dialogue regarding deciding what is important for the district.*” Manager, district A

“*Some few innovations have resulted from this; we might not measure their effect now but maybe in future. For example, we set up an intensive care unit* [for children]*, it is not big, but an area with everything available, and kids are better.” Manager, district B*

### Understanding of evidence in the planning process

Despite having a different understanding of the meaning of evidence, district managers said that evidence was the data they collect for the health department. Some of the respondents understood evidence as having proof about something, or that something works, while others thought that it refers to the use of quantitative data that was collected through Health Management Information Systems or surveys like Lots Quality Assurance Surveys. Others reported that evidence was information that was relevant to establishing the actual situation of the health department irrespective of its source and this could include community members and information collected from other sources, for example, from the political or religious leaders in the district.

“*I think evidence means using data and facts derived from data, so using evidence is somehow connected to data.*” Manager, district A

## Discussion

Findings showed that district managers included in the district health annual work plans approximately half of the activities that were identified to bridge gaps for child survival. There was a slight increase in the number of prioritised activities over the 2012/2013 to 2015/2016 period. However, the proportion of activities included in the annual work plans remained practically the same over the 4-year period. Although procurement and logistics-related activities, such as reproducing guidelines, accounted for the most substantial proportion of planned funding in both districts, there was a wide variation in what those activities included. The variation might be an indication that the districts prioritised activities according to their local needs. Child survival activities accounted for between 4% and 5.5% of the total planned expenditure on health services, with per capita funding of US$ 0.3 in one district and US$ 0.1 in the other. Over the 4 years, donors’ contributions accounted for between 47% and 94% of the funding for child survival activities.

DHMT members acknowledged that the use of district-specific evidence resulted in practical and robust annual work plans and was beneficial to the district health system. This is similar to findings in a study conducted in India, Nigeria and Ethiopia, where district managers viewed the use of locally generated evidence positively [48]. However, in one of the districts, activities that were considered too expensive, and no funds were available, were not prioritised in the work plan. In contrast, in the other district, even those priorities that were unfunded but considered of high priority were included in the work plan and indicated as unfunded. The difference in the action taken showed that the district managers involved in the planning process were not always clear about what should be included in the annual health work plans. Should it be all the activities they consider priorities irrespective of available funding? Or should they prioritise only those activities that can fit within the indicative financing for that financial year? This lack of clarity could be related to the absence of a regional level and the weakened capacity of the MoH to coordinate, support, monitor and supervise the growing number of districts [5], leading to the deterioration of the planning process. Similar findings were documented by Youngkong et al. [19] in a review study on priority-setting in developing countries. In other studies conducted in sub-Saharan countries by Mutale et al. [49] and Henriksson et al. [4], financial constraints affected decision-making and prioritisation of activities despite the use of district-specific evidence.

The DHMT considered the lack of autonomy or decision space a constraint to the use of district-specific evidence. Similar concerns have been documented in other LIC settings like Ghana, Zambia and the Philippines [50–52]. Although being decentralised, most of the priority-setting is still carried out at the central level with districts following the national guidelines from the MoH [7]. District managers also reported difficulties in reallocating funds, indicating limited fiscal space. Donors and other partners contributed most of the funding for child survival activities, although, as was demonstrated in another study in Uganda [18], often the donor agencies have their own priorities, which may not always be those of the districts, for example, the geographical area of implementation of programmes may not necessarily be in the areas that have the most need.

Another consideration while using district-specific evidence was the political nature of decision-making and priority-setting. This is not unique to Uganda, Bryant et al. [53] and Goddard et al. [54] also documented politics as a primary consideration in decision-making processes. Another factor that influenced the use of district-specific evidence was influence from multiple funders/stakeholders, as was also reported in a review study on setting priorities for health interventions by Youngkong et al. [19].

## Methodological considerations

The absence of a standard format for the district annual work plan meant that the work plans varied between the districts and even between the different planning cycles. The variation was not as a result districts identifying context-specific priorities. The difference in formats makes the analysis more difficult to standardise and generalise. As this study was carried out in only two districts that were part of an intervention to improve the use of district-specific evidence in setting priorities in the planning process, results cannot be generalised to all other districts; however, they can be used to inform the planning process in Uganda and countries with similar settings. During the annual health work plan review, analysis of planned activities and not their implementation was done. The analysis also included the proposed expenditure for child survival activities and not the actual spending.

## Conclusions

This study revealed that use of district-specific evidence and a structured process for its utilisation to prioritise activities and make decisions in the planning process at the district level helped systematise an otherwise ad hoc process. By using district-specific evidence, health management teams were able to articulate and advocate for priorities related to child survival. However, the reported limited decision and fiscal space, human resource gaps and inadequate funding did not always allow for the use of district-specific evidence in the planning process. As reflected in the district health annual work plans, districts were heavily dependent on donor funding for child survival activities, which may constrain their ability to use district-specific evidence in the planning process.

The heavy dependence on donor funding raises the question about the usefulness of using district-specific evidence in the reported absence of adequate resources to finance and operationalise the district health annual work plans and the limited decision and fiscal space that the DHMT has to address the gaps identified using the local evidence. Related to this is whether district managers can prioritise activities that reflect the needs of the district as opposed to the interests of the funding partners who provided the bulk of funding for child survival activities.

## Abbreviations

CODES: Community and District Empowerment for Scale-up project
DHMT: District Health Management Team
DHS: District Health System
LIC: low-income country
MoH: Ministry of Health
